# Stimulant treatment profiles predicting co-occurring substance use disorders in individuals with attention-deficit/hyperactivity disorder

**DOI:** 10.1007/s00787-019-01283-y

**Published:** 2019-02-05

**Authors:** Annabeth P. Groenman, Lizanne J. S. Schweren, Wouter Weeda, Marjolein Luman, Siri D. S. Noordermeer, Dirk J. Heslenfeld, Barbara Franke, Stephen V. Faraone, Nanda Rommelse, Catharina A. Hartman, Pieter J. Hoekstra, Jan Buitelaar, Jaap Oosterlaan

**Affiliations:** 1 Clinical Neuropsychology Section, Vrije Universtiteit Amsterdam, Amsterdam, The Netherlands; 2 Center of Child and Adolescent Psychiatry, University Medical Center Groningen, University of Groningen, Accare, Groningen, The Netherlands; 3 Department of Psychology, Leiden University, Leiden, The Netherlands; 4 Departments of Human Genetics and Psychiatry, Radboud University Medical Center, Nijmegen, The Netherlands; 5 Donders Institute for Brain, Cognition and Behaviour, Radboud University, Nijmegen, The Netherlands; 6 Departments of Psychiatry and of Neuroscience and Physiology, SUNY Upstate Medical University, Syracuse, NY USA; 7 Department of Biomedicine, K.G. Jensen Centre for Research on Neuropsychiatric Disorders, University of Bergen, Bergen, Norway; 8 Karakter Child and Adolescent Psychiatry University Center, Nijmegen, The Netherlands; 9 Department of Psychiatry, Radboud University Medical Center, Nijmegen, The Netherlands; 10 Department of Psychiatry, University of Cambridge, Cambridge, UK; 11 Emma Children’s Hospital AMC, Amsterdam, The Netherlands; 12 Department of Pediatrics, VU Medical Center, Amsterdam, The Netherlands; 13 Department of Psychiatry, University Medical Center Groningen, Groningen, The Netherlands

**Keywords:** ADHD, Substance use disorders, Nicotine dependence, Stimulant medication

## Abstract

Adolescents with attention-deficit/hyperactivity disorder (ADHD) are at increased risk of developing substance use disorders (SUDs) and nicotine dependence (ND). It remains unclear whether and how stimulant treatment may affect this risk. We aimed to investigate how stimulant use profiles influence the risk of SUDs and ND, using a novel data-driven community detection analysis to construct different stimulant use profiles. Comprehensive lifetime stimulant prescription data and data on SUDs and ND were available for 303 subjects with ADHD and 219 controls, with a mean age 16.3 years. Community detection was used to define subgroups based on multiple indicators of treatment history, start age, treatment duration, total dose, maximum dose, variability, stop age. In stimulant-treated participants, three subgroups with distinct medication trajectories were distinguished (late-and-moderately dosed, *n* = 91; early-and-moderately dosed, *n* = 51; early-and-intensely dosed, *n* = 103). Compared to stimulant-naïve participants (*n* = 58), the early-and-intense treatment group had a significantly lower risk of SUDs and ND (HR = 0.28, and HR = 0.29, respectively), while the early-and-moderate group had a significantly lower risk of ND only (HR = 0.30). The late-and-moderate group was at a significantly higher risk of ND compared to the other two treatment groups (HR = 2.66 for early-and-moderate, HR = 2.78 for early-and-intense). Our findings show that in stimulant-treated adolescents with ADHD, long-term outcomes are associated with treatment characteristics, something that is often ignored when treated individuals are compared to untreated individuals.

**Electronic supplementary material:**

The online version of this article (10.1007/s00787-019-01283-y) contains supplementary material, which is available to authorized users.

## Introduction

Individuals with attention-deficit/hyperactivity disorder (ADHD) are at increased risk of developing substance use disorders (SUDs) and of starting smoking [1]. Stimulant treatment is the first choice pharmacological treatment of ADHD [2] because it has been proven efficacious in reducing core symptoms of the disorder [3, 4]. In recent years, concerns that stimulant treatment might increase SUDs and smoking in ADHD have been invalidated [5, 6]. One meta-analysis [6] found that stimulant treatment did not affect the development of SUDs or nicotine dependence (ND), whereas the other meta-analysis [5] found a protective effect of stimulants on tobacco use. Possibly, stimulant treatment may have a protective effect in earlier phases of smoking, but not in later stages (i.e., ND). These inconclusive results may be explained by differences in outcome measure severity (smoking vs ND), or indicate unidentified moderators on the development of SUDs and smoking.

Studies have reported earlier initiation of stimulant treatment [7–9] and longer duration of stimulant use [10] as possibly enhancing the protective effect on the development of SUDs; however, other studies did not replicate these findings [11, 12]. Preclinical studies suggest that the brain may be more sensitive to the effect of stimulants during adolescence (i.e., critical or sensitive age periods) [13]. A recent study predicted substance-related behavior from both age of treatment onset and duration of treatment, and found that short, late-onset stimulant treatment increased the risk of SUDs. Unfortunately, this study did not account for ADHD-severity, a factor related to both stimulant treatment and the risk of SUDs [14], and looked at both factors separately. In contrast, earlier onset of use [7–9], longer duration [10], and higher treatment continuity [5] could have positive long-term effects on SUDs and smoking. Previous studies have looked at these factors individually, but to the best of our knowledge, no studies have investigated these factors in concert to assess their joint predictive power on SUDs and smoking.

The objective of this study is to investigate how stimulant use profiles are associated with the risk of SUDs and ND. Here, we used a novel technique of community detection to identify distinct subgroups of patients with ADHD based on multiple indicators of stimulant treatment history (i.e., stimulant use profiles). This technique has previously been used in a partly overlapping, but smaller sample where we, successfully predicted increased brain activation during reward receipt in those treated early and intensely [15], in a brain area important in the development of SUDs and smoking. With this study, we build on the previous results comparing stimulant-treated subjects with stimulant-naïve and controls [16]. Start age, treatment duration, total dose, maximum dose, variability, and stop age were derived from highly detailed individual pharmacy transcripts. We hypothesized that adolescents with ADHD who started treatment at younger age, and were treated longer and at a more stable dose, would have a lower risk of SUDs and ND compared to adolescents with a history of later, lower dose, and variable treatment.

## Methods

### Participants

Participants were selected from the Dutch part of the International Multicenter ADHD Genetics (IMAGE) study [17]. Data collection of IMAGE took place between 2003 and 2006. ADHD families were recruited from outpatient clinics and included at least one child aged 5–17 years with combined-type ADHD and at least one biological sibling regardless of ADHD diagnosis. Exclusion criteria applying to all participants included autism, epilepsy, IQ < 70, brain disorders, and any single gene disorder associated with externalizing behaviors that might mimic ADHD (e.g., fragile-X). Additionally, control families were recruited from primary and high schools from similar geographical regions as participating ADHD families.

For the current study, participants were followed-up on an average 4.2 (SD 0.7) years after the enrolment in the study (data collection 2008–2009). Ethical approval for the study was obtained from National Institute of Health registered ethical review boards and local ethical review boards and written informed consent was obtained from all participants and/or their parents. During this follow-up 511 participants with ADHD and 220 control participants above the age of 12 participated (84.17% of the original ADHD sample and 76.38% of the original control sample).

The current paper includes all participants above the age of 12 at follow-up with ADHD and with information on substance use and pharmacy data (*n* = 303, of which 58 participants were stimulant-naïve) at follow-up. No differences were found between those participants with ADHD successfully followed-up and those lost to follow-up on ADHD-severity, impairment, age (*p* > 0.24 for all measures), sex (*p* = 0.73), ODD (*p* = 0.85) or CD (*p* = 0.58). A total of 219 control subjects with no history or family history of psychiatric disorders were available to study differences between participants with ADHD (trajectory groups) and controls. Most commonly prescribed stimulants were immediate-release methylphenidate (87.5%), extended-release methylphenidate (70.1%), and dexamphetamine (8.6%); other non-stimulant medication was commonly prescribed, and could thus not be excluded, but sensitivity analysis will be done. Most commonly prescribed non-stimulant medications were low-dose atypical antipsychotics, mostly risperidone (21.7%), atomoxetine (14.8%), anxiolytics/benzodiazepines (6.1%), and antidepressants (7.3%).

### Measurements

#### Diagnostic assessment

##### Assessment of ADHD, oppositional defiant disorder, and conduct disorder at baseline

At study entry, participants from ADHD families were screened for ADHD, using standard procedures of the IMAGE project (see supplement). In short, DSM-IV diagnoses [18] and symptom counts (i.e., severity) for ADHD, conduct disorder (CD) and oppositional defiant disorder (ODD) were based on the Parental Account of Childhood Symptoms interview in combination with the Long Version of the Conners Parent and the Teacher Rating Scales. All subjects with ADHD met full DSM-IV criteria for ADHD.

##### Substance use disorder at follow-up

At follow-up, assessment of SUDs (both alcohol and drug use disorder), and smoking were obtained using the Diagnostic Interview Schedule for Children (DISC-IV-P), Alcohol Use Disorders Identification Test (AUDIT), Drug Abuse Screening Test–20 (DAST), and Fagerström Test for Nicotine Dependence (FTND). SUDs were defined as a positive score on either the DISC-IV-P, AUDIT, or DAST (for details, see supplement). As previous meta-analyses [5, 6] found contrasting results concerning smoking and stimulant treatment, we operationalized smoking in two ways: (1) daily smoking of less than 10 cigarettes, and (2) ND (a score of 6 or higher on the FTND, or a positive score on the tobacco module of the DISC-IV-P).

##### Stimulant medication use

**Fig. 1:**
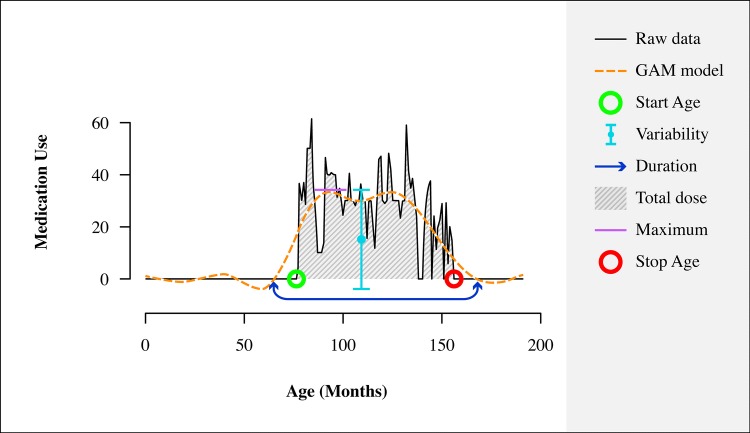
Example of a single subjects’ data. Data from a single subject with a fitted GAM model. *GAM* generalized additive models. Duration of use, maximum dose and variability of dose are based on the GAM model. Medication use = average monthly daily dose

Lifetime pharmacy transcripts were collected at follow-up. On an average, pharmacy transcripts covered 69.1% of lifetime. When pharmacy data were missing, self-report data were used (also see Supplement). Pharmacy data were used to reconstruct treatment trajectories per participant with high temporal resolution. Stimulant trajectories in mg per day were constructed for each day between dates of birth and follow-up. To reduce effects of high frequency dose changes (inherent to the high temporal resolution), we derived smoothed treatment trajectories as well using a generalized additive model (GAM) in R [19, 20]. The following measures were extracted from either the raw treatment trajectories or the fitted GAM models: start age (raw trajectory); treatment duration relative to age (duration [GAM model] divided by age minus the earliest start age within the sample, i.e., 2.3 years); total dose [raw trajectory] relative to age; maximum dose (GAM model); variability (SD) of the dose (GAM model); and stop age (raw trajectory) (see Fig. 1 for an example of a single subjects data).

### Statistical analyses

The six variables describing individual medication trajectories were entered in a Louvain community detection algorithm in R3.2.2 [21, 22] to identify distinct subgroups of stimulant-treated patients with ADHD based on treatment profiles [23]. The algorithm classifies participants such that similarities within groups as well as differences between groups are maximized. The modularity (*Q*) quantifies the degree to which separation between subgroups is achieved (*Q* of zero indicates no subgroups, and a *Q* of one perfect segregation between subgroups). The algorithm iterates until *Q* no longer increases, indicating the solution will not improve with further iterations. Stimulant-naïve subjects were manually added as separate subgroup.

Comparisons between medication subgroups were performed using SPSS 22 (IBM SPSS Statistics version 22). Differences between groups in gender, age, IQ, ADHD-severity, and CD comorbidity were examined using analysis of variance and Chi square tests. We examined differences in the development of SUDs and smoking (daily smoking vs. ND) in the subgroups using cox proportional hazard models. The models used age at first substance or nicotine use as the survival time for the cases (classified as having an SUD and/or daily smoking/ND) and current age as the time of censoring for the non-cases. Correction for clustered (family) data was done using robust standard errors [24].

Sensitivity analyses were performed following significant results, to assess the effect of known confounders (i.e., SES, ADHD-severity, IQ, CD and the use of non-stimulants) and to see how medication groups differed from controls, (these analyses are described in full in the supplement). Matched group analyses (based on age and hyperactive symptoms) were performed to rule out the effect of these variables.

## Results

### Community detection

**Fig. 2:**
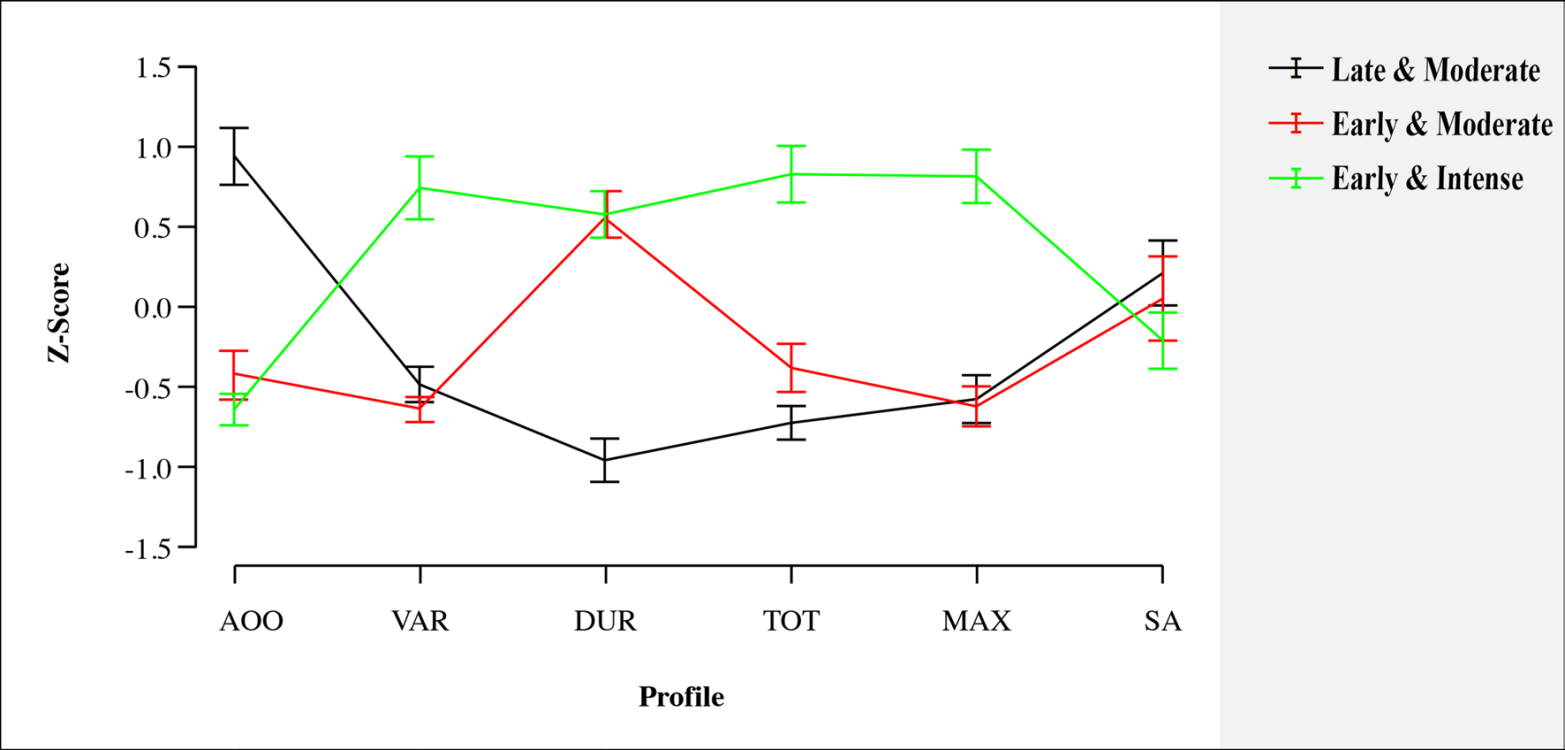
Community detection outcomes. This figure depicts the three medication subgroups that were defined by the community detection algorithm: (1) a late-and-moderate use group characterized by a late onset of treatment, short duration, and moderate total dose and maximum use, (2) a early-and-moderate use group characterized by a young onset age, a long duration of use, and a late offset of treatment age, and (3) early-and-intense use group characterized by a young onset of treatment age, a variable trajectory of medication use with a long duration, high total dosage, high maximum dosage and early age at treatment offset. *AOO* stimulant medication offset, *VAR* variability of dose (SD), *DUR* duration of use, *TOT* total dose, *MAX* maximum dose, *SA* stop age

The community detection procedure yielded three medication subgroups of stimulant-treated patients (Fig. 2; *Q* = 0.61). The fourth medication subgroup was manually added, namely the stimulant-naïve group. Bootstrap analyses (non-parametric bootstrap with 1000 replications) showed high stability of the three-class solution, which was identified in 94.8% of the runs, with mean *Q* = 0.60 (SD = 0.02; see Supplement). The largest group (*n* = 103) was characterized by young onset of treatment age, variable trajectory of medication use with a long duration, high total and high maximum dose, and young age at treatment offset (also see Table 1 and Fig. 2 for characteristics of the groups). We referred to this group as ‘early-and-intense use’ subgroup. The second group (*n* = 91) was characterized by late-onset age of treatment, short duration, and moderate total dose and maximum use, referred to as ‘late-and-moderate use’ subgroup. The third group (*n* = 51) was characterised by young treatment onset age, long duration, and late offset age, referred to as ‘early-and-moderate use’ subgroup. The fourth subgroup was comprised of the 58 stimulant-naïve subjects.

**Table 1:** Subject Characteristics

	Stimulant-Naïve (*n *= 58)	Late-and-moderate use (*n *= 91)	Early-and-moderate use (*n *= 51)	Early-and-intense use (*n *= 103)	Controls (*n *= 219)	Test-value	*P* value	Contrasts
Gender, n males (%)	44 (75.9)	68 (73.9)	42 (82.4)	89 (86.4)	88 (40.2)	*χ*^2^ = 90.06	<0.001	4 < (0 = 1=2 = 3)
Age at follow-up	17.22 (2.68)	16.77 (2.52)	16.09 (2.11)	15.28 (2.07)	16.34 (2.52)	*F* = 7.79	<0.001	0 = 1=2 = 4, 3 < (0 = 1=4), 3 = 2
Age at baseline	12.60 (2.72)	12.29 (2.51)	11.53 (2.03)	10.75 (2.17)	12.60 (2.65)	*F* = 11.23	<0.001	0 = 1=2 = 4, 3 < (0 = 1=4), 3 = 2
IQ	98.4 (15.22)	99.53 (12.96)	96.97 (13.01)	100.95 (13.65)	105.56 (9.52)	*F* = 6.41	<0.001	4 > (0 = 1=2 = 3)
Hyperactive symptoms	7.08 (2.23)	7.83 (1.4)	7.88 (1.47)	8.25 (1.15)	–	*F* = 6.42	<0.001	3 > 0, 0 = 1=2, 1 = 2=3
Inattentive symptoms	8.04 (1.24)	7.90 (1.69)	8.20 (0.98)	8.07 (1.07)	–	*F* = 0.79	0.50	0 = 1=2 = 3
ODD, *n* (%)	16 (30.2)	25 (33.3)	24 (50.0)	35 (43.2)	–	*χ*^2^ = 5.79	0.12	0 = 1=2 = 3
CD, *n* (%)	10 (18.9)	17 (22.7)	7 (14.9)	15 (18.5)	–	*χ*^2^ = 1.17	0.76	0 = 1=2 = 3
SES	12.22 (2.75)	11.20 (1.94)	11.26 (2.39)	11.32 (2.04)	12.58 (2.66)	*F* = 8.03	0.001	0 = 1=2 = 3,0 = 4, 4 > (1,2,3)
Age of onset^b^		11.47 (2.39)	7.73 (1.32)	7.09 (1.53)		*F* = 145.08	<0.001	1 > (2 = 3)
Stop age^b^		15.73 (2.58)	15.31 (2.42)	14.66 (2.34)		*F* = 4.79	0.009	1 > 3 1 = 2, 2 = 3
Duration^c, d^		0.73 (0.06)	0.87 (0.05)	0.87 (0.7)		*F* = 150.45	<0.001	
Variability^d^		98.93 (101.15)	70.49 (51.73)	334.08 (204.62)		*F* = 82.26	<0.001	3 > (1 = 2)
Maximum dose in mg^d^		23.93 (14.61)	22.62(9.81)	53.35(17.89)		*F* = 114.01	<0.001	3 > (1 = 2)
Cummulative use^c^		5.70(4.05)	8.54 (4.37)	18.51 (7.41)		*F* = 129.04	<0.001	1 < 2<3
SUDs, *n* (%)	19 (32.8)	23 (25.8)	12 (23.5)	8 (7.8)	26 (11.9)			
Daily Smoking^a,^*n* (%)	23 (39.7)	28 (30.8)	14(27.5)	28 (27.2)	40 (18.3)			
Nicotine Dependence, *n* (%)	11 (19.0)	14 (15.7)	3(5.9)	5 (4.9)	6 (2.7)			

0 = stimulant-naïve subgroup, 1 = late-and-moderate use subgroup, 2 = early-and-moderate use subgroup, 3 = early-and-intense use subgroup, 4 = controls, SES = socioeconomic status (based on average years of parents’ education). ODD, CD, and ADHD symptoms were measured at baseline. Pairwise comparisons were performed with Tukey with equal variances or Dunnett’s T3 when variances were unequal

*SUDs* substance use disorders

^a^Daily smoking = daily smoking of at least 1 cigarette per day

^b^In years

^c^Corrected for age of possible use, ^d^ derived from the GAM model

Characteristics of the four subgroups are given in Table 1. There were no differences between the four medication subgroups in percentage of males, CD, IQ, or number of inattentive symptoms at baseline. The early-and-intense use subgroup was significantly younger and had more hyperactive-impulsive symptoms at baseline compared to the other medication subgroups. Subgroups of subjects were selected from the empirically derived medication subgroups to yield four groups equivalent in sample size (*n* = 51), age, and hyperactivity-impulsivity (see Supplement for additional details). The medication groups were matched post hoc on age and hyperactivity/impulsivity symptoms at baseline (information on exact selection procedures can be found in the Supplement).

The stimulant-treated subgroups did not differ in percentage of anxiolytics/benzodiazepines or antidepressants prescribed (*p *> 0.05), but the early-and-moderate use subgroup was prescribed atomoxetine more often compared to the early-and-intense use subgroup (24.3% vs. 9.8%), and the early-and-moderate subgroup was prescribed more atypical antipsychotics compared to the late-and-moderate use subgroup (33% vs. 17.6%).

### Substance use disorder

**Fig. 3:**
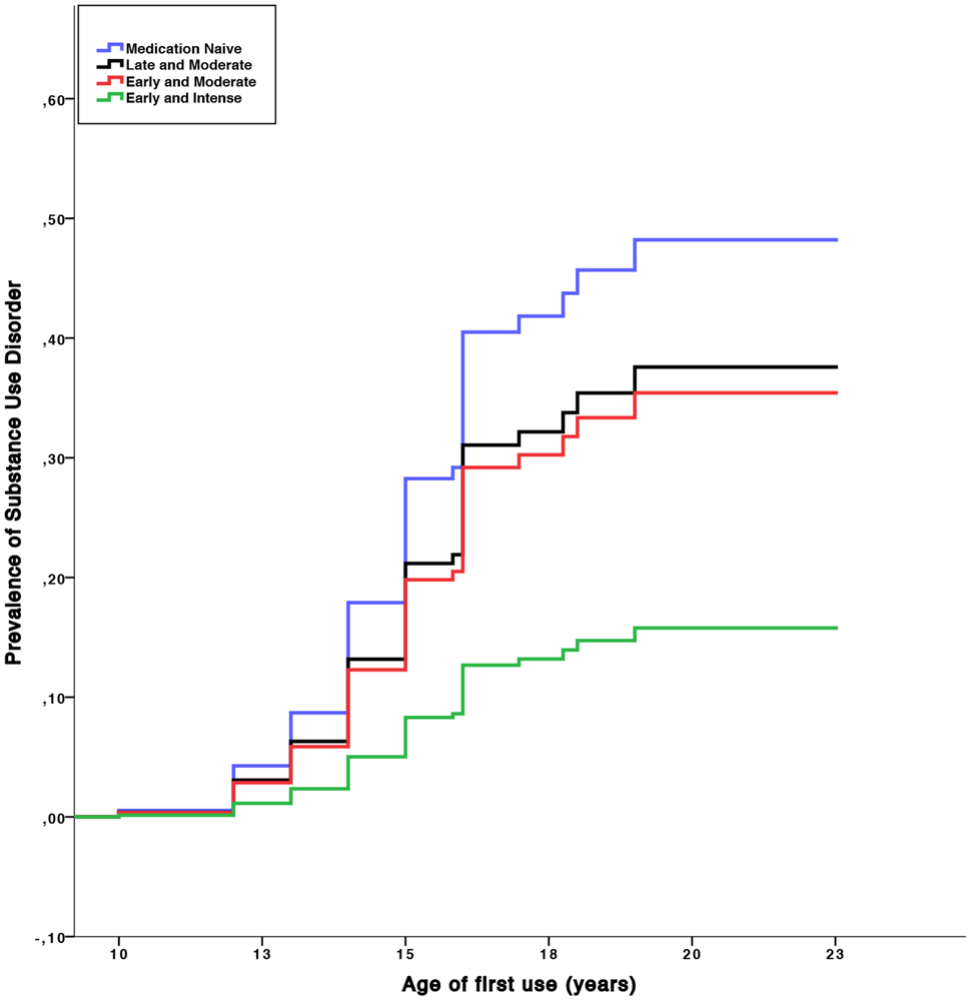
Cumulative lifetime risk for any substance use disorder. One minus survival curve estimated with cox proportional hazard model for development of SUDs (any alcohol or drug use disorder) in subjects with ADHD with age of first substance use on the *x* axis

At follow-up, the medication subgroups differed in the number of participants with SUDs (Wald *χ*^2^ = 25.06, *p *< 0.001; see Table 2 and Fig. 3). The early-and-intense use subgroup was at the lowest risk of developing SUDs compared to the three other subgroups, but no differences were found between the other subgroups. Sensitivity analyses with matched groups (matched on age and hyperactivity/impulsivity symptoms) confirmed that the lower risk for SUDs found in the early-and-intense subgroup was not due to age (also see supplementary Table S2 and Figure S1). Sensitivity analyses showed that the difference in subgroups in SUDs was not due to SES, CD, IQ, ADHD-severity or non-stimulants. Furthermore, sensitivity analyses showed that the early-and-intense subgroup was at comparable risk of developing SUDs to controls, but the no stimulant group (HR 2.03, 95% CI 1.53–2.68), late-and-moderate group (HR 1.65, 95% CI 1.13–2.41), and the early-and-moderate group (HR 1.74, 95% CI 1.40–2.16) were at significantly higher risk compared to controls (also see Table S2).

**Table 2:** Hazard ratios for the analyses comparing the medication subgroups

	Late-and-moderate use vs. naïve		Early-and-moderate use vs. naïve		Early-and-intense use vs. naïve		Late-and-moderate use vs. early-and-intense use		Early-and-moderate use vs. early-and-intense use		Late-and-moderate use vs. early-and-moderate use	
	HR	*P*	HR	*P*	HR	*P*	HR	*P*	HR	*P*	HR	*P*
SUDs	0.74	0.19	0.73	0.16	**0.28**	**<0.001**	**2.70**	**<0.001**	**2.66**	**<0.001**	1.01	0.96
Daily smoking^a^	0.84	0.36	0.92	0.70	1.23	0.26	0.68	0.04	0.75	0.17	0.91	0.67
Nicotine dependence	0.81	0.34	**0.30**	**0.016**	**0.29**	**0.001**	**2.78**	**0.009**	1.04	0.94	**2.66**	**0.045**

Daily smoking = daily smoking of at least 1 cigarette. Bold numbers indicate significance at *p *< 0.05

^a^No significant group effect

### Smoking

#### Daily smoking

**Fig. 4:**
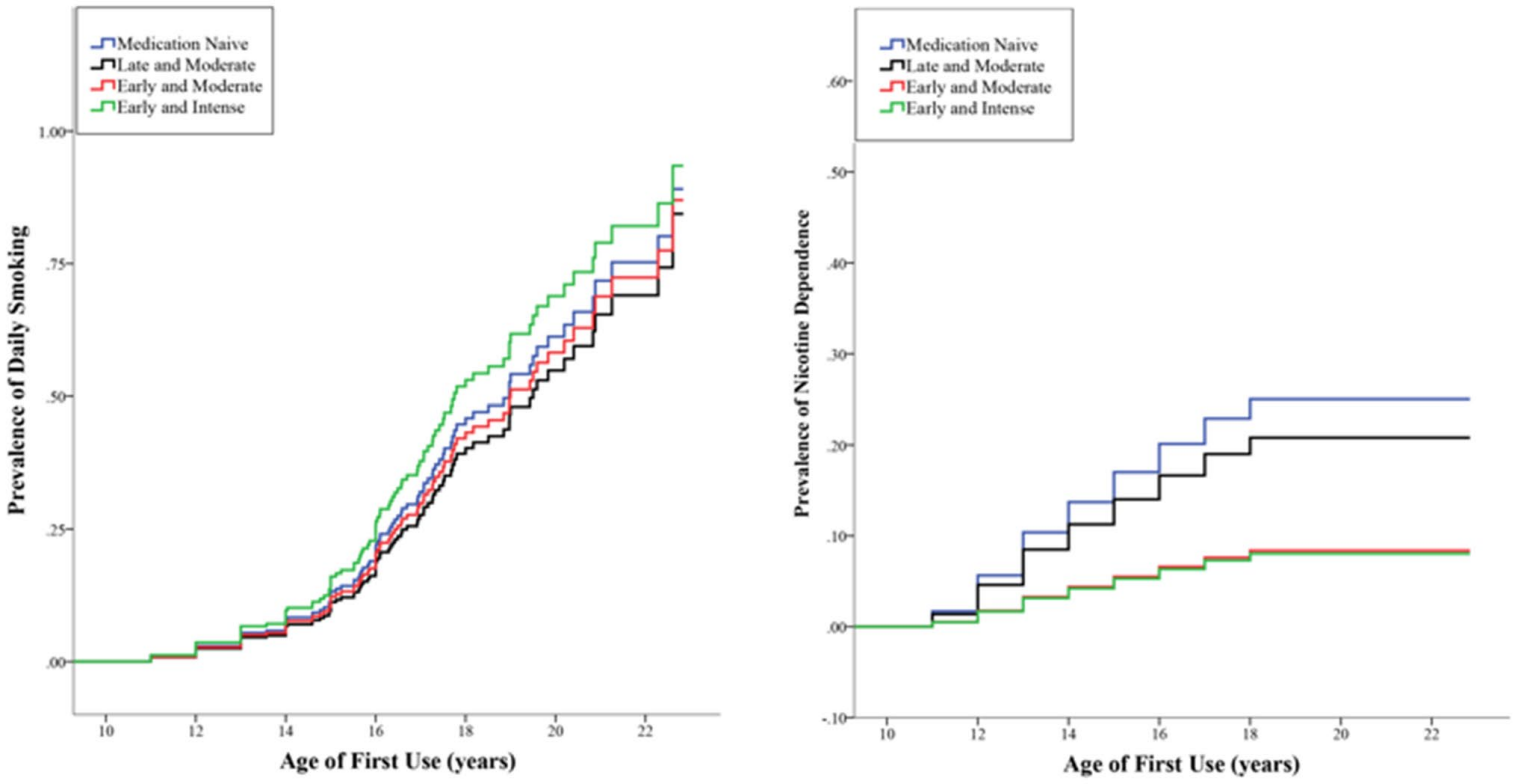
Cumulative lifetime risk for smoking. One minus survival curve estimated with cox proportional hazard model for development of smoking in subjects with ADHD with age of first cigarette use on the *x* axis. Left panel: daily smoking, right panel: nicotine dependence

No differences were found between any of the medication subgroups in the risk of daily smoking (Wald *χ*^2^ = 4.71, *p *= 0.19; see Table and right panel of Fig. 4).

#### Nicotine dependence

Significant differences between the medication subgroups were found in the risk of developing ND (Wald *χ*^2^ = 14.98, *p *= 0.002; see Table 2 and Fig. 4). The early-and-intense use and early-and-moderate use subgroups were at lower risk of developing ND compared to the late-and-moderate use and stimulant-naïve subgroups. The early-and-intense use and the early-and-moderate use subgroups did not differ in their risk, neither did the late-and-moderate use nor the stimulant-naïve subgroups. Sensitivity analyses confirmed that the lower risk for nicotine dependence found in the early-and-intense subgroup was not due to age (also see supplementary Table S2 and Figure S1). Furthermore, it was shown that effects were not dependent on SES, CD, IQ, ADHD-severity or non-stimulants. We found that the early-and-moderate group was at higher risk compared to healthy controls (HR 3.16, 95% CI 1.63–6.14). The late-and-moderate (HR 10.21, 95% CI 6.99–14.92) and the stimulant-naïve groups were also at higher risk from controls (HR 9.91, 95% CI 7.26–13.52) (a full description of the sensitivity analyses can be found in the supplement).

## Discussion

We aimed to examine the association between stimulant medication and the development of SUDs and smoking in individuals with ADHD using community detection to construct stimulant use profiles from highly detailed pharmacy records. This allowed us to look at stimulant treatment history in a new manner. We hypothesized that adolescents with ADHD who started treatment at younger age and were treated longer at a more stable dose, would have a lower risk of SUDs and smoking compared to adolescents with a history of later, lower dose and variable treatment. We confirmed our hypothesis and found that those individuals with ADHD who received treatment at a young age and with a high dose, were at a lower risk of developing SUDs and ND, but those who received treatment at a later age with a lower dose were not. This shows that, when looking at stimulant treatment effects, multiple indicators of stimulant medication use should be taken into account.

The current study significantly advances prior studies by being the first to look at multivariate profiles of medication use using highly detailed pharmacy records, as opposed to prior studies looking at a global measure of medication use (yes/no) using self-report scales. Our findings are in line with prior studies looking at indicators of medication use reporting that treatment duration [14] and age of treatment onset [7, 9] affect the development of SUDs and ND. In addition to these treatment characteristics, our data suggest that cumulative dose, maximum dose, and dose variability also play a role in the development of addictive disorders in ADHD. More specifically, confirming our hypothesis, we found that multivariate profiles characterized by a young start age of medication, a high maximum and cumulative dose are associated with lower risks of SUDs and ND in individuals with ADHD.

Our findings regarding smoking showed a distinction between daily smoking and ND. Whereas the early-and-intense group (characterized by a young onset of treatment age, a variable trajectory of medication use with a long duration, high total dosage, high maximum dosage and early age at treatment offset) and early-and-moderate group (characterized by a young onset age, a long duration of use, and a late offset of treatment age) were at lowest risk of developing ND, the risk of daily smoking was unaffected by stimulant use profile. Furthermore, these findings show the need to take severity of nicotine use into account in future studies. A possibility could be that early in the trajectory of ND, stimulant use does not have an effect, but in the later phases (i.e., ND) it does. However, this seems unlikely, as previous studies have suggested a delay in onset of substance-related disorders as a consequence of stimulant use, with much larger effects of stimulant use on the development in adolescence [11, 25, 26] than in adulthood [27, 28]. However, to make conclusive inferences a later follow-up of the current sample is necessary.

In studies of long-term medication effects such as ours, that are inevitably observational, one should be wary of potential confounding by unmeasured variables (i.e., endogeneity). As an example, unmeasured parental characteristics rather than stimulant treatment may account for some of the differences regarding SUD and ND between treatment groups. Similarly, we cannot rule out the possibility that the early-and-intense use and the early-and-moderate use subgroups differed from the late-and-moderate use group with regard to treatment response or factors associated with MPH response (e.g., genetic predispositions), which in turn may drive the association with SUDs and ND. Treatment response was not assessed in the current study. One may argue, however, that treatment response is most likely associated with treatment duration rather than with age of treatment onset. We recommend future studies of long-term stimulant outcomes to take treatment response into account.

The current study has several strengths. First, we introduce a novel approach of looking at stimulant treatment history that allows integrated analysis of multiple related treatment parameters. This data-driven approach resulted in distinct and ecologically valid subgroups, that had predictive validity with regard to important long-term outcomes. Second, we had access to extensive and highly detailed pharmacy records for the majority of our patients with ADHD. Third, unlike many previous studies of tobacco use, we distinguished between smoking and ND and found that indeed the effects of stimulant treatment on these two outcomes are not the same. Some limitations should be noted as well. Long-term outcomes of stimulant medication can only be studied using naturalistic longitudinal studies, that are inevitably at risk for endogeneity, making inferences about causality impossible. Second, we did not have the opportunity to distinguish between different SUDs, or SUDs of different severity, in a similar fashion as we did for smoking and ND, as the numbers of drug use disorders were low in our sample. While the exactness of pharmacy records were very high detailed and gave us the possibility to use community detection, we cannot assure that medications picked up from the pharmacy were actually taken by the individual. Furthermore, our sample is a clinical sample, and we can only draw conclusion on those subjects in clinical practice. As is common with clinical samples, our ADHD sample contains more males than our controls. While we statistically corrected for this, this could have clouded our results. We feel that our results are meaningful since our ADHD sample is representative of those seeking help for their problems. Additionally, comorbidities are common in those seeking help, and while disruptive behavioral problems are most common, other comorbidities are frequent, and should be taken into account in future studies. Especially since treatment effects can be different in different comorbid subgroups (e.g., [29]). Of note, factors associated with early-and intense-treatment, such as higher ADHD-severity and higher levels of CD, are both also associated with a higher risk of SUDs. Interestingly, this group was associated with a lower risk of SUDs and ND. Furthermore, we did not have further information on treatment response and tolerability of treatment, and for this reason we recommend future studies to take these into account. Finally, no data were available regarding psychosocial interventions; if the reduced risk of SUDs reported in this study is associated with a reduction in symptoms of ADHD (caused by the use of stimulants), one would expect to find other treatments with the potential to lower ADHDs core symptoms, such as effective psychosocial interventions, to have a protective effect as well.

## Conclusion

In conclusion, we add to current literature by showing that, in stimulant-treated patients with ADHD, there are distinct trajectories of medication use that are differentially related to the risk for SUDs and ND. There is evidence to support the idea that untreated ADHD is related to worse outcomes than treated ADHD [30–32]. Here, we corroborate this evidence, and expand on this by showing that a medication profile characterized by a late start age, low dose, and low duration of stimulant treatment also has worse outcomes compared to medication profiles with an early start age, high or moderate dose, and long duration of stimulant treatment. We want to emphasize the importance of optimal titration and proper monitoring of stimulant medication in the treatment of ADHD; stimulant treatment should be at adequate dosages to reduce the risk of SUDs and ND as negative long-term outcomes associated with ADHD. Furthermore, our results show an association between starting stimulant treatment at an early age and a reduced risk of developing negative long-term outcomes. Importantly, long-term outcomes of stimulant-treated adolescents with ADHD are associated with treatment characteristics, something that is often ignored when treated individuals are compared to untreated individuals.

## Electronic supplementary material

Below is the link to the electronic supplementary material.

Supplementary material 1 (DOCX 316 kb)

## Abbreviations

ADHD: Attention-deficit/hyperactivity disorder
CD: Conduct disorder
GAM: General additive model
HR: Hazard ratio
IQ: Intelligence quotient
MPH: Methylphenidate
ND: Nicotine dependence
ODD: Oppositional defiant disorder
SD: Standard deviation
SES: Socioeconomic status
SUDs: Substance use disorders
95% CI: 95% Confidence interval
