# Supplementary material for: Stimulant treatment profiles predicting co-occurring substance use disorders in individuals with attention-deficit/hyperactivity disorder
Source: Eur Child Adolesc Psychiatry. 2019 Feb 5;28(9):1213–22. doi: 10.1007/s00787-019-01283-y (PMC6751155; doi:10.1007/s00787-019-01283-y)
Supplement: Supplementary file 1 — Supplementary material 1 (DOCX 316 kb) [file 787_2019_1283_MOESM1_ESM.docx]

**Supplementary Methods**

**Diagnostic criteria**

***Assessment of ADHD, oppositional defiant disorder and conduct disorder at baseline.*** Baseline measures included the Parental Account of Childhood Symptoms PACS; ^1^ interview. The PACS is a semi-structured, standardized, investigator-based interview developed to provide an objective measure of child behavior. A trained interviewer administered the PACS to parents, who were asked for detailed descriptions of their child’s typical behavior in a range of specified situations for an exact description of the interview procedure, we refer to ^2^. These included the Long Version of Conners’ Parent (CPRS-R:L), and Teacher Rating Scale CTRS-R:L; ^3^. A standardized algorithm was applied to the PACS to derive each of the 18 DSM-IV ADHD-symptoms, providing operational definitions for each behavioral symptom. These were combined with items that were scored 2 (pretty much true) or 3 (very much true) in the teacher-rated Conners ADHD subscales (L, M, and N) to generate the total number of hyperactive-impulsive and inattentive symptoms of the DSM-IV symptom list ^4^. A standardized algorithm was applied to combine symptom count on the PACS and CTRS-R:L, both providing operational definitions of each of the 18 behavioral symptoms defined by the DSM-IV. Situational pervasiveness was defined as symptoms occurred within two or more different situations as assessed with the PACS interview, as well as the presence of one or more items scored 2 or higher or more from the ADHD scale of the CTRS-R:L for a full description of the algorithm procedure also see,^5^. The PACS sections dealing with disruptive behavior (ODD and CD) are structured similarly to the ADHD section, except that symptoms are not evaluated across multiple situations. The PACS assesses all DSM-IV ^4^ ODD and CD symptoms. Symptom ratings were dichotomized with absence of a symptom coded as 0 and presence of symptom as 1, and then summed separately for ODD and CD symptoms. Two categorical measures of disruptive behavior encompassing ODD and CD were defined. The first measure defined ODD according to the DSM-IV criteria based on information from the PACS and the CPRS-R:L. The second measure defined CD according to the DSM-IV criteria using a standardized algorithm applied to the PACS. Subjects with ODD were not allowed to score positive for CD, while subjects with CD were allowed to score positive for ODD. In addition to the interview, rating scales were used to quantify ADHD and ODD severity.

***Substance use at follow-up.***

A parental report of SUD was obtained using the SUD module of the Diagnostic Interview Schedule for Children DISC-IV-P; ^6^. The DISC-IV-P was administered by telephone, and scored with a computer-algorithm to derive DSM-IV-defined SUD diagnoses. Age of first substance use was also assessed in the interview. A number of questionnaires was completed by participants. The Alcohol Use Disorders Identification Test AUDIT; ^7^ was used to identify self-reported alcohol dependence. Scores on the AUDIT may range from 0-40. A score of 9 or higher was used to define alcohol abuse, and a score of 13 or more in girls and 15 or more in boys was used as a cut-off to define alcohol dependence ^7^. The Drug Abuse Screening Test–20 DAST; ^8^ was used to assess drug use disorders. Scores on this questionnaire may range from 0–20. A cut-off of 5 was used to identify possible drug use disorders ^8^. The Fagerström test for ND FTND; ^9^ was used to assess ND. Scores on this questionnaire may vary between 0 and 10. A cut-off of 6 was used to identify ND ^9^. Age of first nicotine use was also assessed in this questionnaire. All measures ask the participant about the previous 12 months. All participants were provided with a personal return envelope to increase trust, and ensure confidentiality of sensitive information.

A best-estimate diagnosis of SUD was considered present if either alcohol or drug use disorder was present in the subject. A best-estimate diagnosis of alcohol use disorder was considered present if either scores on the AUDIT (self-report) or DISC-IV-P alcohol module (parent-report) met criteria stated above. A best-estimate diagnosis of drug use disorder was considered present if either the DAST (self-report) or DISC-IV-P marihuana module or other drugs module (parent-report) met criteria stated above.

Furthermore, two measures of smoking were defined based on the DISC-IV-P and the FTND namely daily smoking (i.e. daily smoking of at least one cigarette assessed with FTND) and nicotine dependence (i.e. either a score of 6 or higher on the FTND (self-report), or a positive score on the tobacco module of the DISC-IV-P (parental-report)).

**Missing data.**

On average, pharmacy transcripts covered 69.1% of lifetime, and complete *lifetime* pharmacy data was available for 28.9% of participants. If missing data for a participant entailed less than 60 time points (months), data was imputed using several approaches: missing data before the start age was imputed with a dose of 0 (in 2 cases with a mean length of 32.5 months), missing data between two points of the *same* value were filled with this value (in 47 cases, with a mean of 37.15 months), gaps smaller than 12 months and a difference between the first missing and last missing time-point, were filled with a linear fill (in 5 cases, with a mean of 6.2 months). This led to 54 imputes on the data.

**Bootstrapping Analyses.**

Using non-parametric bootstrapping, the community detection algorithm was rerun 1000 times. The algorithm produced a three-class solution on 948 of the 1000 runs (94.8%), a 2-class solution on 38 out of 1000 runs (3.8%), and a 4 class solution on 14 out of 1000 runs (1.4%). In the three-class solution, on average, the largest class contained 42.7% of participants (SD=4.6%) the second largest class contained 37.5% of participants (SD=3.4%) and the smallest class contained 19.3% of participants (SD=7.1%). In the solution presented in the paper the largest class contained 41.9% of all subjects the second largest 37.4% and the smallest 20.7% of subjects, resembling the average solution of the bootstrapping method. Moreover, the confidence intervals are narrow and standard deviations are small, indicating stable solutions across runs. On average Q was .6040 (.0198), closely resembling the Q reported in the paper, a plot of Qs over all runs can be seen in Figure S3. From this we can conclude that the three-class model used is a highly stable solution.

**Age matching**

The rule out possible age effects in our results, and because increasing age increases the risk for later SUDs and ND; we created age-matched groups for sensitivity analyses. We matched our medication using groups to our smallest medication group (early-and-moderate) on age (< 1 year age difference). It was not possible to match the medication naïve group; we kept the number of subjects the same (i.e. n=51) as the smallest medication group, and used an age trim (i.e. all subjects above the age of 20.3 were removed), leading to equal age groups. Furthermore, we matched controls to the smallest medication use group in age, but also on gender (also see Table S1).

**Sensitivity analyses**

Sensitivity analyses included: (1) a re-run using age-matched subgroups to rule out the effect of age. Following significant results, the following analysis steps were undertaken: (2) we compared the medication subgroups and controls on SUDs and smoking using age-matched groups, (3) and we assessed the effects of known confounders (i.e. SES, ADHD-severity, IQ, CD, and use of co-medication) in the age-matched groups by adding them as covariates to the model (analyses were not corrected for ODD, as previous analyses revealed no effect of ODD on SUDs and ND in the current sample^10^).

**Supplement Results**

**Substance use disorders.**

***Posthoc tests for substance use disorder.*** Sensitivity analyses (1) confirmed that the lower risk for SUDs found in the early-and-intense subgroup was not due to age (also see supplementary Table S2 and Figure S1). In step two of the Sensitivity analyses it was found that the early-and-intense subgroup was at comparable risk of developing SUDs to controls (HR=.56, 95%CI=.24-1.29), but the no stimulant group (HR=2.03, 95%CI=1.53-2.68), late-and-moderate group (HR=1.65, 95%CI=1.13-2.41), and the early-and-moderate group (HR=1.74, 95%CI=1.40-2.16) were at significantly higher risk compared to controls (also see Table S2). In step three it was found that the original results were not dependent on SES, CD, IQ, ADHD severity or co-medication. The early-and-intense group was at significantly lower risk of developing SUDs compared to the early-and-moderate (HR= 8.75, 95%CI=6.53-11.71), late and moderate (HR= 7.30, 95%CI=47-11.37), and stimulant naïve group (HR=9.75, 95%CI=6.91-13.76) after correction for SES, CD, IQ, ADHD-severity and co-medication. In this model there was a significant effect of CD (HR=2.44, 95%CI=1.45-4.10) and IQ (HR=1.03, 95%CI=1.01-1.04) on the development of SUD, but no significant effect of total symptom count (HR= 1.00, 95%CI=.93 -1.09), co-medication (HR=1.08, 95%CI=.88-1.33), or SES (HR= 1.02, 95%CI=.88-1.17).

**Nicotine dependence**

***Sensitivity analysis for nicotine dependence.*** Step 1 of the sensitivity analyses revealed that results were not dependent on age (also see supplementary Table S2 and Figure S2). In step two we found that the early-and-moderate group differed in their risk to healthy controls (HR=3.16, 95%CI=1.63-6.14). The late-and-moderate (HR=10.21, 95%CI=6.99-14.92) and the medication naïve group also differed from controls (HR=9.91, 95%CI=7.26-13.52). In step three we found that the original results did not chance after taking known confounders into account. In this model there was a significant effect of CD on the development of ND was found (HR= 2.29, 95%CI=1.35-4.89), a significant effect of total symptom count (HR= .85, 95%CI=.78-.94) but not of SES (HR=1.03, 95%CI=.83-1.27), IQ (HR=.99, 95%CI=.97-1.01), or co-medication (HR=1.01, 95%CI=.82-1.46).

Supplementary Tables & Figures

Table S1. Subject characteristics, based on age-matched groups

|  | Naïve  (*n*=51 ) | Late and Moderate  (*n*=51) | Early and Moderate  (*n*=51) | Early and Intense  (*n*=51) | Healthy Controls (n=51) | Test-value | *p*-Value | Contrasts |
| --- | --- | --- | --- | --- | --- | --- | --- | --- |
| Gender (n males (%)) | 40(78.4) | 38(74.5) | 42(82.4) | 45(88.2) | 41(80.4) | *χ^2^*=3.39 | .50 | 0=1=2=3=4 |
| Age | 16.67(2.36) | 16.18(2.15) | 16.08(2.11) | 16.00(1.95) | 16.30(2.03) | *F*=.76 | .55 | 0=1=2=3=4 |
| IQ | 97.11(15.26) | 99.92(12.93) | 96.97(13.01) | 99.11(13.01) | 106.20(9.47) | *F*=4.13 | .003 | 4>(0=1=2=3) |
| SUDs n (%) | 14 (27.5) | 18 (35.3) | 15 (29.4) | 12 (23.5) | 6 (11.8) |  |  |  |
| Daily Smoking^a ,^ n (%) | 18 (35.3) | 16 (31.4) | 18 (35.3) | 14 (27.5) | 8 (15.7) |  |  |  |
| Nicotine Dependence, n (%) | 14 (27.5) | 18 (35.3) | 15(29.4) | 12 (23.5) | 6 (11.8) |  |  |  |
| Hyperactive symptoms | 6.94(2.30) | 7.91(1.29) | 7.88(1.47) | 7.82(1.37) | - | *F*=3.67 | .013 | 0=1=2=3 |
| Inattentive symptoms | 8.00(1.29) | 7.84(1.27) | 8.20(.98) | 7.95(1.14) | - | *F*=.79 | .499 | 0=1=2=3 |
| ODD (%)^a^ | 13(28.3) | 14(34.1) | 24(50.0) | 19(47.5) | - | *χ^2^*=6.16 | .104 | 0=1=2=3 |
| CD (%)^a^ | 9(19.6) | 10(24.4) | 7(14.9) | 6(15.0) | - | *χ^2^*=1.72 | .63 | 0=1=2=3 |
| SES | 12.12(2.71) | 10.79(1.80) | 11.25(2.39) | 11.26(2.01) | 12.65(2.44) | *F=4.04* | .003 | 0=1=2=3, 1<4, 4=0=2=3 |

*Note.* Naïve = Medication Naïve*,* 0 = No Stimulant Treatment, 1 = Late and moderate, 2= early and moderate, 3= early and intense. ^a^ Data was not available for all subjects. Controls were also matched on gender.

Table S2. Odds ratios for the risk of substance use disorders and smoking for age-matched groups

|  | Main effect | | Late and Moderate vs. Naïve | | Early and Moderate  vs. Naïve | | Early and Intense vs. Naïve | | Late and Moderate  vs. Early and Intense | | Early and Moderate  vs. Early and Intense | | Late and Moderate  vs. Early and Moderate | |
| --- | --- | --- | --- | --- | --- | --- | --- | --- | --- | --- | --- | --- | --- | --- |
|  | Wald *χ^2^* | *p-*value | HR | *p* | HR | *p* | HR | *p* | HR | *p* | HR | *p* | HR | *p* |
| SUD | **8.54** | **.036** | .85 | .50 | .42 | .42 | **.28** | **.007** | **3.04** | **.024** | **3.11** | **.009** | .98 | .94 |
| Daily Smoking 1-10^a^ | 2.06 | .56 | 1.19 | .42 | .89 | .56 | 1.10 | .70 | 1.09 | .71 | .81 | .37 | 1.33 | .17 |
| Daily Smoking ≥10^a^ | 7.40 | .06 | .93 | .78 | .47 | .03 | .59 | .12 | 158 | .17 | .80 | .58 | 1.96 | .039 |
| Malleable ND | **8.27** | **.041** | 1.44 | .08 | .82 | .36 | 1.10 | .67 | 1.31 | .17 | .74 | .22 | **1.77** | **.009** |
| ND | **15.14** | **.002** | 1.04 | .89 | **.32** | **.004** | **.22** | **.04** | **4.78** | **.035** | **4.6** | **.04** | **3.22** | **.003** |

*Note*. Naïve= meidcation naïve*,* SUD= substance use disorder, Daily smoking 1= daily smoking of at least 1 cigarette, Daily Smoking 10= daily smoking of at least 10 cigarettes, Malleable ND = ND above 18 years old, and daily smoking below 18 years. Bold figures indicate significance at *p*<.05. ^a^No signficant group effect.

Figure S1. Cumulative risk for substance use disorders for age-matched groups

*
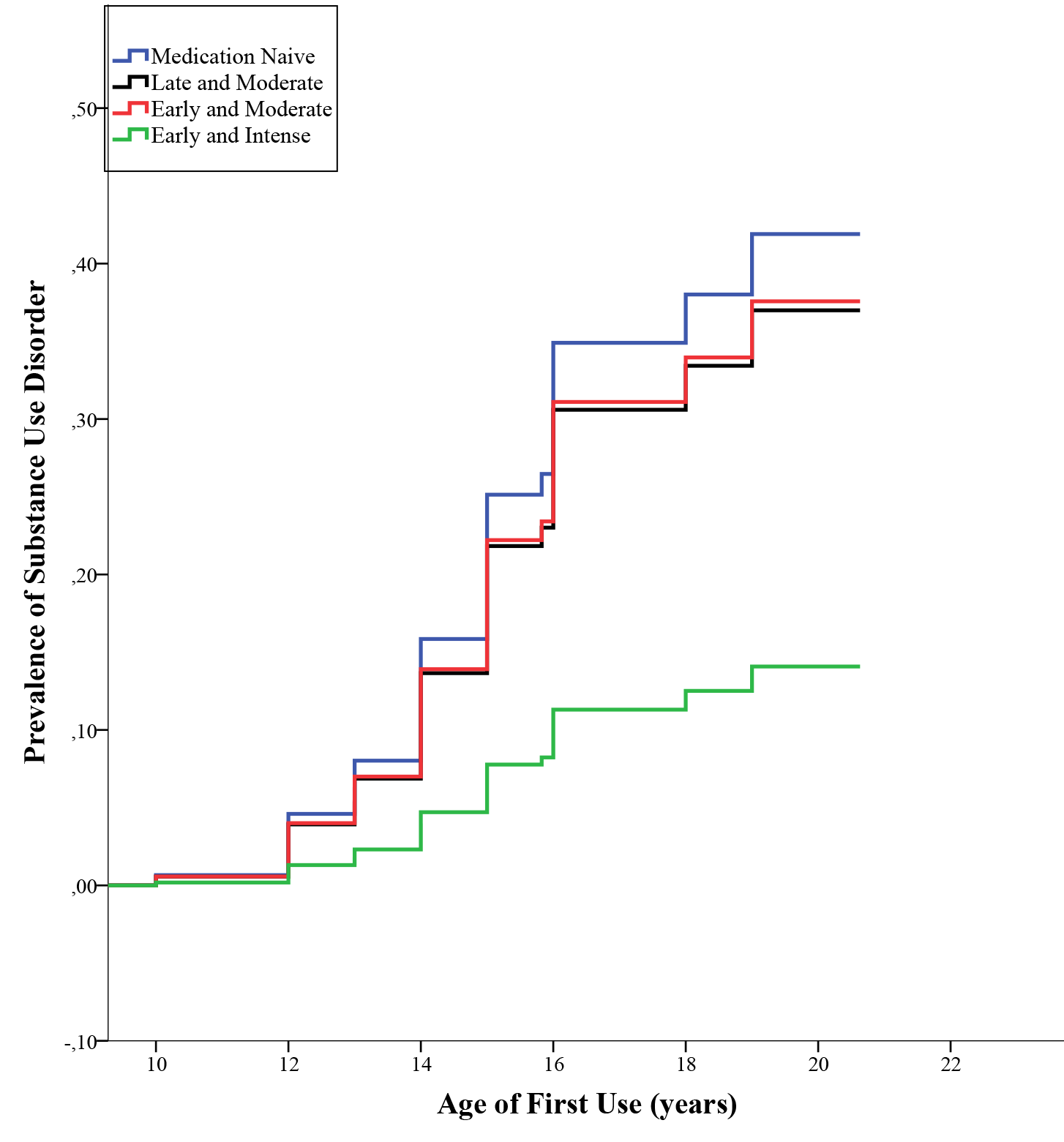
*

*Note.* One minus survival curve estimated with cox proportional hazard model for development of SUDs (either alcohol or drug use disorder) in subjects with ADHD with age-matched groups.

Figure S2. Cumalative lifetime risk of smoking for age-matched groups
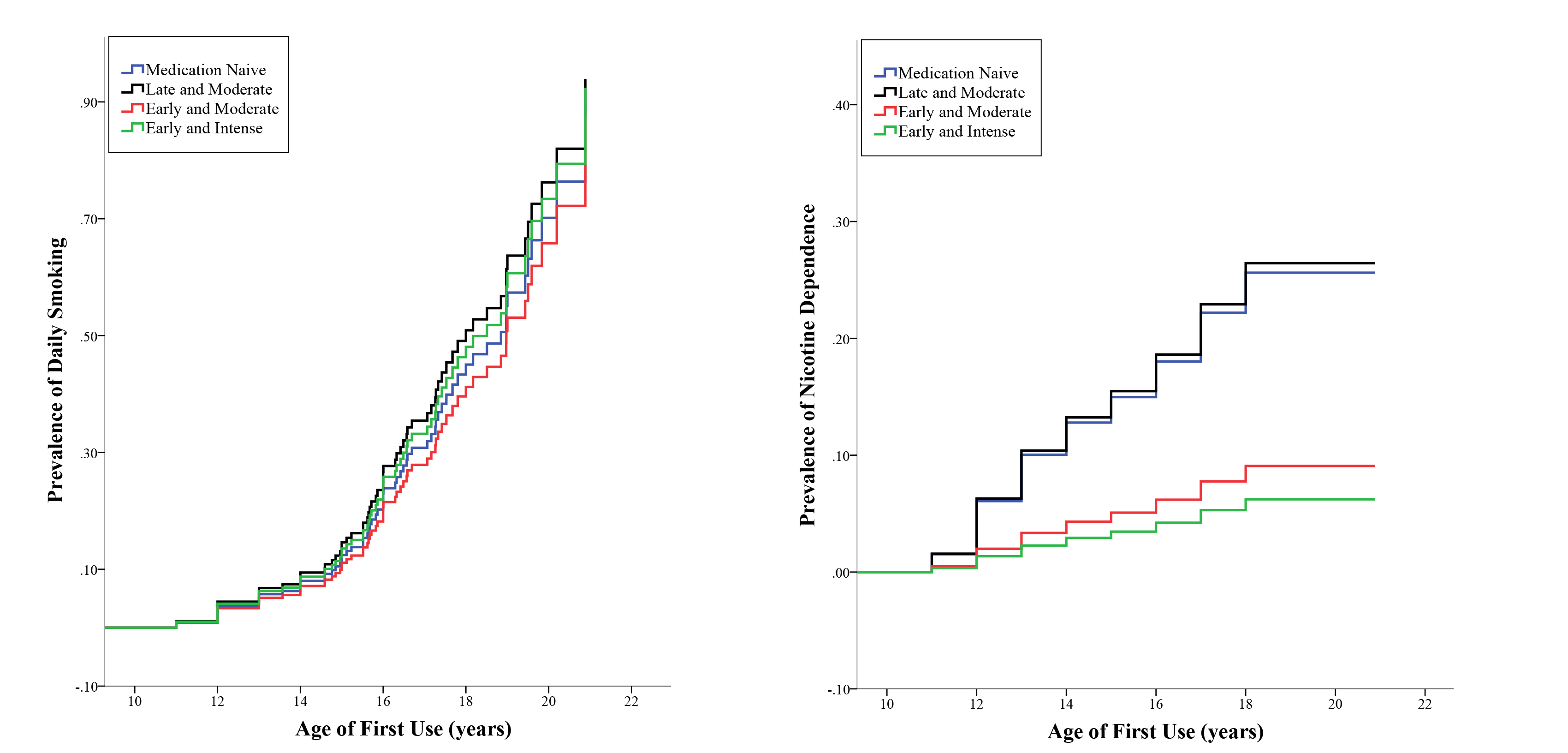


*Note.* One minus survival curve estimated with cox proportional hazard model for development of smoking in subjects with ADHD with age matched groups. Left panel: Daily smoking, right panel: nicotine dependence.

Figure S3. Stability of Q over 1000 runs


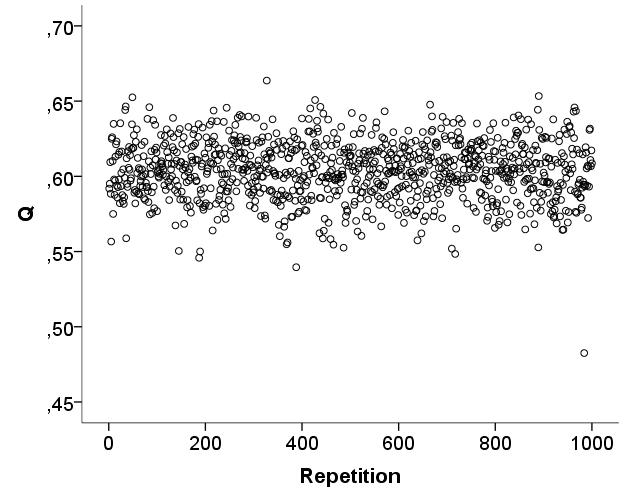


1. Chen W, Taylor E. Parental Account of Children's Symptoms (PACS), ADHD Phenotypes and its Application to Molecular Genetic Studies. 2006.

2. Brookes K, Xu X, Chen W, et al. The analysis of 51 genes in DSM-IV combined type attention deficit hyperactivity disorder: association signals in DRD4, DAT1 and 16 other genes. *Mol Psychiatry.* 2006;11(10):934-953.

3. Conners CK, Sitarenios G, Parker JD, Epstein JN. The revised Conners' Parent Rating Scale (CPRS-R): factor structure, reliability, and criterion validity. *J Abnorm Child Psychol.* 1998;26(4):257-268.

4. American Psychiatric Association. *Diagnostic and statistical manual of mental disorders [DSM-IV-TR].* Washington, DC: American Psychiatric Association; 2000.

5. Rommelse NNJ, Oosterlaan J, Buitelaar J, Faraone SV, Sergeant JA. Time reproduction in children with ADHD and their nonaffected siblings. *Journal of the American Academy of Child and Adolescent Psychiatry.* 2007;46(5):582-590.

6. Shaffer D, Fisher P, Lucas CP, Dulcan MK, Schwab-Stone ME. NIMH Diagnostic Interview Schedule for Children Version IV (NIMH DISC-IV): description, differences from previous versions, and reliability of some common diagnoses. *J Am Acad Child Adolesc Psychiatry.* 2000;39(1):28-38.

7. Saunders JB, Aasland OG, Babor TF, de la Fuente JR, Grant M. Development of the Alcohol Use Disorders Identification Test (AUDIT): WHO Collaborative Project on Early Detection of Persons with Harmful Alcohol Consumption--II. *Addiction.* 1993;88(6):791-804.

8. Gavin DR, Ross HE, Skinner HA. Diagnostic validity of the drug abuse screening test in the assessment of DSM-III drug disorders. *Br J Addict.* 1989;84(3):301-307.

9. Heatherton TF, Kozlowski LT, Frecker RC, Fagerstrom KO. The Fagerstrom Test for Nicotine Dependence: a revision of the Fagerstrom Tolerance Questionnaire. *Br J Addict.* 1991;86(9):1119-1127.

10. Groenman AP, Oosterlaan J, Rommelse N, et al. Substance Use Disorders in Adolescents with Attention Deficit Hyperactivity Disorder: A Four-Year Follow-up Study. *Addiction (Abingdon, England).* 2013.
